# Spectroscopic Characteristics and Speciation Distribution of Fe(III) Binding to Molecular Weight-Dependent Standard Pahokee Peat Fulvic Acid

**DOI:** 10.3390/ijerph19137838

**Published:** 2022-06-26

**Authors:** Yaqin Zhang, Chang Liu, Yuxia Li, Liuting Song, Jie Yang, Rui Zuo, Jian Li, Yanguo Teng, Jinsheng Wang

**Affiliations:** 1 College of Water Sciences, Beijing Normal University, Beijing 100875, China; zhangyaqin129@163.com (Y.Z.); liuchangslgc@163.com (C.L.); hhuliyuxia@163.com (Y.L.); zr@bnu.edu.cn (R.Z.); lijian@bnu.edu.cn (J.L.); ygteng@bnu.edu.cn (Y.T.); wangjs@bnu.edu.cn (J.W.); 2 Engineering Research Center of Groundwater Pollution Control and Remediation, Ministry of Education of China, Beijing 100875, China

**Keywords:** Pahokee Peat fulvic acid, dissolved organic matter, iron, complexation, ultraviolet–visible, fluorescence, Donnan Membrane Technique

## Abstract

Peat-derived organic matter, as powerful chelators, is of great significance for the transport of Fe to the ocean and the enhancement of dissolved Fe. However, the iron binding capacity of molecular weight (MW)-fractionated dissolved organic matter is variable, due to its structure and composition heterogeneity. In this work, we used the standard Pahokee Peat fulvic acid (PPFA) as an example, and investigated the spectroscopy properties and Fe(III) binding ability of PPFA and different molecular weight fractions by UV–Vis absorbance and fluorescence spectroscopy and the Donnan Membrane Technique (DMT). The results showed binding sites for Fe(III) at the 263 nm and >320 nm regions in differential absorbance spectra. Upon increasing the iron concentration to 18.00 μmol·L^−1^, the critical binding capacity was exceeded, which resulted in a decrease in absorbance. Fe(III) was found to prefer to bind to humic-like components, and ultraviolet humic-like fluorophores displayed stronger binding strength. High molecular weight PPFA fractions (>10 kDa) possessed more aromatic and hydrophobic components, displayed a higher degree of humification, and exhibited higher metal binding potential. Furthermore, the speciation analysis and stability constant (^c^K) were calculated using Donnan membrane equilibrium. The correlation between ^c^K values and PPFA spectral properties demonstrated that aromaticity, hydrophobicity, molecular weight and humification degree were crucial indices of PPFA–Fe(III) affinity. Significantly, the humification degree, represented by HIX, showed the strongest correlation (r = 0.929, *p* = 0.003), which could be used to estimate the binding strength. This study provides further understanding of the complexation mechanism of iron and DOM in the peat environment and identifies the considerable effect of molecular weight.

## 1. Introduction

Iron is one of the most abundant elements in the surface environment and plays an irreplaceable role in the life activities of organisms [1,2]. However, the bioavailable iron only accounts for about 1% of total iron in the environment, and iron deficiency limits the marine primary productivity, especially in the high nutrient low chlorophyll regions (HNLC) [3]. Recent evidence suggests that peatlands play an important role in the mobility of iron and that terrigenous dissolved organic matter (DOM), originally derived from peat, acts as a chelator for iron [4]. Due to the strong binding ability of ligands (fulvic acid and humic acid), the content of dissolved iron will be greatly increased, and the amount of dissolved iron thus contributed to the oceans is two orders of magnitude higher than the “average world” rivers [3,5,6]. Many studies have proven that >99% of the dissolved Fe in the ocean is iron–organic complexes, which improve the bioavailability of microorganisms for Fe(III) [7,8]. Because of the protection by organic ligands, the high conditional stability constants for Fe(III)–humic complexes determined in seawater suggest that iron can be migrated over long distances by rivers [9]. In addition, the extensive degradation of original wetlands led to a significant decrease in peatlands, and thus to a disturbance in the global circulation of biologically active iron [10].

Previous studies have shown that the molecular weight of humic substances is also a vital factor in the transport of iron from peatlands to the open ocean [11,12,13]. The operationally defined “total dissolved Fe” (<0.45 μm) fraction of natural water may contain the colloidal form (10 kDa–0.45 μm) and truly dissolved form (<10 kDa). Thus, composition heterogeneity caused by molecular weight (MW) is also an essential contributor. It is difficult to elucidate the dynamics of the dissociation and protonation processes in DOM and the interaction mechanism because of the complexity of its structural features, composition and molecular weight distribution. The formation of these DOM-metal may be influenced by concentration, pH, ionic strength, and the salt present [14,15]. Changes in the above factors all affect the complexation of Fe(III) and DOM, so further study of the interaction of peat-derived DOM and iron is essential to identify binding characteristics.

Various optical techniques have been applied to explain the interaction between metals and DOM, such as ultraviolet–visible absorption spectroscopy (UV–Vis) and three-dimensional excitation-emission matrix fluorescence spectroscopy (3D EEM). For example, it is found that the absorbance at certain wavelengths can help to indicate properties of the DOM and quantify metal ions binding onto the DOM [16]. Moreover, differential and ln-transformation absorbance spectra have been recently developed to quantify the interactions between metal ions in the reactive binding sites in the DOM [17,18]. Furthermore, 3D EEM measurements are employed to provide useful information on the structural properties of DOM, and the Stern–Volmer model based on the quenching of the fluorescence intensity of DOM can help to explain the binding ability [19,20]. Fluorescence regional integration (FRI) is a quantitative technique for analyzing DOM by integrating the volume beneath each EEM region, using all the wavelength-dependent fluorescence of EEMs [21,22].

The Donnan Membrane Technique (DMT) is an efficient and sensitive method to determine free metal ion concentrations, which uses a cation exchange membrane to separate the sample solution (donor) and acceptor containing only non-colloidal cationic species [23]. At present, Cu^2+^, Zn^2+^, Pb^2+,^ Cd^2+^, Al^3+^, Ni^2+^, Ag^+^ and Eu^3+^ have been successfully measured by DMT, but the utility of this method for Fe^3+^ ion is still lacking [24,25,26].

In this paper, we investigated iron binding to standard Pahokee Peat fulvic acid (PPFA) using absorbance and fluorescence techniques. Bulk PPFA was fractionated into low molecular weight (LMW, <10 kDa) and high molecular weight (HMW, 10 kDa–0.45 μm) fractions based on the ultrafiltration procedure and HMW components were fractionated into 10–100 kDa, 100–1000 kDa and 1000 kDa–0.45 μm. The changes in absorbance and fluorescence of the PPFA–Fe complexation with varying Fe(III) concentrations and different molecular weight were examined to determine the effect of PPFA binding features. The speciation and binding capacity of Fe(III) after complexation equilibrium were characterized by Donnan Membrane Technique (DMT). In addition, changes in the selected representative absorbance and fluorescence parameters were compared with the complexation constant based on DMT to identify the significance and suitability of the selected absorbance and fluorescence parameters.

## 2. Materials and Methods

### 2.1. Sample Preparation

All chemicals mentioned in this paper were all guaranteed reagent grade, unless otherwise mentioned. All solutions were prepared using Milli-Q water (18.2 MΩ cm^−1^, Millipore Corp., Burlington, MA, USA). The sample of standard Pahokee Peat fulvic acid II (PPFA, 2S103F) was purchased from the International Humic Substance Society (IHSS), and the selected chemical characteristics are listed in Table S1. The Pahokee peat is obtained from typical agricultural peat soil of the Florida Everglades, which were formed in organic deposits of freshwater marshes [27]. Stock solutions (Fe^3+^) were prepared using Fe(NO_3_)_3_ from Aldrich Chemical Company (Milwaukee, WI). Sr(NO_3_)_2_ (0.01 M) was prepared as a background electrolyte. NaOH (0.1 M) and HNO_3_ (0.1 M) were used for the regulation of pH.

Bulk PPFA was obtained by dissolving in Sr(NO_3_)_2_ (0.01 M) shaken at room temperature for 24 h without light and 0.45 μm filter through a cellulose acetate filter membrane. The molecular weight (MW) fractionated samples of PPFA were obtained by a Labscale tangential flow ultrafiltration system (Pellicon System, Millipore Co., Ltd.) using 1000 kDa, 100 kDa, and 10 kDa membranes. Before ultrafiltration, the membrane was cleaned with 5 mol·L^−1^ NaOH (5 M) and ultrapure water. PPFA bulk solution was graded into 1000 kDa–0.45 μm, 1000 kDa, 10–100 kDa and less than 10 kDa, named P1, P2, P3 and P4, respectively. The ultrafiltration results are shown in Table S2. All DOMs mentioned above were diluted to a dissolved organic carbon (DOC) concentration of 50 mg L^−1^ using Sr(NO_3_)_2_, measured by a TOC-analyzer (Vario, Elementar). All dissolved organic samples were used and prepared immediately, stored in the dark at 4 °C until analysis, and used within 7 days.

### 2.2. Titration Experiments and Complexing Model

The binding characteristics of iron with bulk and MW-fractionated PPFA solutions were investigated by adding Fe(NO_3_)_3_ to a series of samples. Iron concentrations in solutions were determined by a spectrophotometric method, with final concentrations ranging from 1.75 to 18.00 μmol·L^−^^1^ for bulk PPFA experiments. For molecular weight fractions, the iron concentration was prepared at 10.00 μmol·L^−1^. The pH values of the titrated solutions were adjusted by HNO_3_ and maintained at 2.00 ± 0.05 to avoid oxide precipitation [28]. After metal addition, the mixed solutions were shaken at room temperature for 48 h in the dark to ensure the full reaction for later uses, as detailed in Table S3. Eventually, UV–Vis absorption and three-dimensional fluorescence excitation-emission matrix (3D EEM) of the solutions were conducted.

In order to quantitatively describe the complex of Fe(III)–PPFA, assuming that Fe^3+^ formed a 1:1 complex with PPFA, the modified Stern–Volmer equation is applied as follows [29]:

(1) $$\frac{F_{0}}{F_{0}-F}=\frac{1}{f\;\cdot {\;K}_{M}\;\cdot {\;C}_{M}}+\frac{1}{f}$$

where $F_{0}$ and F are the fluorescence intensities of the corresponding peaks of DOM samples without and with Fe addition; f represents the fraction of the initial fluorescence that corresponds to the binding fluorophore, which is accessible to quencher; $K_{M}$ is the conditional stability constant, calculated by plotting $F_{0}/F_{0}\;–\;F$ against $1/C_{M}$; $C_{M}$ is the metal concentration.

### 2.3. Spectral Properties and Analysis

#### 2.3.1. UV–Vis

The UV–Vis absorption spectra were measured using a Shimadzu UV-2600 spectrometer, with wavelengths ranging from 200 nm to 800 nm at 0.5 nm increments. To perform concentration-independent comparisons, the DOC-normalized absorbance was obtained by dividing the intensity at the corresponding wavelength by the DOC concentration of the sample. The specific ultraviolet absorbance at 254 nm (SUVA_254_) and 260 nm (SUVA_260_) were calculated as 100 times the ratio of A(*λ*) at each wavelength to the concentration of DOC, resulting in a dimension of m^−1^/(mg-C L^−1^). The SUVA_254_ is especially related to CO and C=C bonds of aromatic C, representing a strong correlation with aromaticity and the hydrophobic fraction of DOM, as well as a useful proxy for DOM molecular weight [30]. SUVA_260_ indicates the proportion of hydrophobic substances in the DOM, and the higher the value, the stronger the hydrophobicity [31]. The E_250_/E_365_ is the ratio of UV–Vis absorbance at 250 nm and 365 nm correlated with the humification degree and the molecular weight of DOM. The higher value represents the lower molecular weight and higher humification degree [32]. Similarly, the E_250_/E_203_ characterized as the ratio of absorbance at 250 nm and 203 nm implies the number of hydroxyl, carboxyl, carbonyl and ester substituents on the aromatic rings [33].

The differential and ln-transformed differential absorption spectra are calculated to reveal the change of absorbance spectra during the Fe-DOM interacting process using the equation as follows [34]:

(2) $${DA}_{\lambda }=\frac{A_{\lambda ,i}-{\;A}_{\lambda ,ref}}{\mathrm{DOC}\cdot L}$$

(3) $${\mathrm{DLn}A}_{\lambda }={\mathrm{Ln}A}_{\lambda ,i}-\;{\mathrm{Ln}A}_{\lambda ,ref}$$

where $A_{\lambda ,i}$ and $A_{\lambda ,ref}$ are the DOM absorption measured at the wavelength *λ* for any selected condition and any applicable reference. The spectrum recorded without the addition of iron is used as the reference. Moreover, the DS_325_–_375_ (differential spectral slope of ln-transformed absorbance in the range of wavelengths 325–375 nm) and DLn_350_ (differential logarithm of DOM absorbance at 350 nm) are proved to estimate the binding of the DOM to heavy metals [35].

#### 2.3.2. Three-Dimensional EEM

The three-dimensional fluorescence excitation-emission matrix (3D EEM) was measured by a Hitachi F-7000 fluorescence spectrometer, with an excitation wavelength (EX) of 220–500 nm and emission wavelength (EM) of 300–600 nm at 5 nm increments. The slit bandwidth was 5 nm, and the scanning rate was 2400 nm min^−1^. The spectrum of ultrapure water was recorded at intervals of 10 analyses, and interference of the first and second scatter peaks was eliminated. Three fluorescence indices, including FI (fluorescence index), BIX (biological index) and HIX (humification index), were calculated to gain further DOM properties. The FI is defined as the ratio of fluorescence intensities between EM 450 and 500 nm at EX 370 nm. FI provides information on the source of DOM and the contribution of aromatic and non-aromatic DOM [36], with FI below 1.4 and above 1.9 divided into terrestrial and microbial sources [37]. BIX is the ratio of EM intensity at 380 nm divided by the EM at 430 nm, while EX at 310 nm, which is applied to determine the contribution of the DOM autochthonous source. BIX values ranging from 0.8 to 1.0 represent a predominantly autochthonous origin of biological or microbial origin, whereas BIX of <0.6 indicates little amount of organic matter of an autochthonous origin [37]. The normalized HIX is defined as the integrated area under the EM 435–480 nm, divided by the peak area of the sum of total intensities in the (300–345) plus (435–480) nm regions [38]. The higher values correspond to the greater humified molecules and more stable DOM [39].

To further clarify the interaction between iron and DOM, differential fluorescence is calculated using the following equation:

(4) $$\Delta {\mathrm{EEM}=\mathrm{EEM}}_{ref}-{\;\mathrm{EEM}}_{i}$$

where ${\mathrm{EEM}}_{ref}$ and ${\mathrm{EEM}}_{i}$ are the fluorescence intensity for reference (for example, bulk and fractionated PPFA without iron) and any selected condition (such as, iron concentrations or PPFA molecular weight) respectively. ΔEEM refers to the fluorescence spectra involved in the reaction.

On this basis, fluorescence regional integration (FRI) analysis was used to further obtain the details of fluorescence changes caused by PPFA and iron interaction. According to the previous studies, the EEM fluorescence is separated into five regions by excitation/emission (EX/EM) wavelengths [22,29]. Regions I and II are connected with simple aromatic proteins, such as tyrosine with peaks at shorter excitation wavelengths (<250 nm) and shorter emission wavelengths (<350 nm). Region III is associated with fulvic-like substances in the range of EX (220–250 nm) and EM (380–500 nm). Region IV (EX 250–280 nm, EM 200–380 nm) and Region V (EX 250–400 nm, EM 380–500 nm) are related to soluble microbial by-product-like substances and humic-like organic matter [40]. The integral volume of the fluorescence region Φ_i_ is calculated by applying the following equation:

(5) $$\phi_{i}=\overset{\;}{\underset{\mathrm{ex}}{\int }}\overset{\;}{\underset{\mathrm{em}}{\int }}{I(\lambda }_{\mathrm{ex}}\lambda_{\mathrm{em}}{)d\lambda }_{\mathrm{ex}}{d\lambda }_{\mathrm{em}}$$

The normalized excitation-emission area ($\phi_{i,n}$) and the fluorescent percentage ($P_{i,n}$) are calculated as follows:

(6) $$\phi_{i,n}=MF_{i}\phi_{i}\;\phi_{T,n}=\sum_{i=1}^{5}\phi_{i,n}\;P_{i,n}=\phi_{i,n}/\phi_{T,n}\times 100\%$$

where ${\mathrm{MF}}_{i}$ is the multiplication factor of each region equal to the reciprocal of the fraction of the projected Ex/Em area. $\phi_{T,n}$ is the integrated standard volume of the total fluorescence area.

### 2.4. Donnan Membrane Technique (DMT)

The DMT device used in this experiment was designed based on a previous study [41]. The cation exchange membrane applied in DMT is Nafion 117 from the DuPont Company, which separates the donor cell and the acceptor cell and allows only cations to pass through. In our experiments, membranes with diameters of 2.9 cm were soaked for 24 h in a mixture containing 10% methanol, 10% nitric acid and 80% Milli-Q water to remove the adsorbed cations. They were then rinsed and soaked in Milli-Q water for 4 h and then in fresh background solutions (0.01 M Sr(NO_3_)_2_). The background solution was replaced 3 times within 24 h. A custom-machined cell used for DMT was made from polychlorotrifluoroethylene plastic. The whole system was carefully cleaned with concentrated nitric acid, Milli-Q water and 0.01 M Sr(NO_3_)_2_ before operation.

The above samples of interest with a volume of 500 mL were used as donor solutions, containing Sr(NO_3_)_2_, Fe(NO_3_)_3_ and DOM. The acceptor solution consisted of 30 mL 0.01 M Sr(NO_3_)_2_ at pH 2. The volume of the donor solution is much larger than that of the acceptor solution, which ensures that the loss caused by the diffusion of Fe ions is negligible relative to the total Fe content, and preserves the chemical equilibrium of the donor side. As shown in Table S6, the Donnan equilibrium was reached after 96 h completely, which was consistent with previous studies [42,43]. When reaching equilibrium, ‘free’ Fe, organic bound Fe and inorganic bound Fe existed on the donor side, while only ‘free’ Fe was contained on the acceptor side. Fe and Sr on both sides were determined by ICP-AES (SPECTRO ARCOS EOP). Considering the effect of the ionic strength differences between the acceptor and donor solutions, the free Fe concentration (Fe_donor_) on the donor side could be calculated by the following equation [41]:

(7) $${\left(\frac{{\mathrm{Fe}}_{\mathrm{donor}}}{{\mathrm{Fe}}_{\mathrm{acceptor}}}\right)}^{\frac{1}{3}}={\left(\frac{{\mathrm{Sr}}_{\mathrm{donor}}}{{\mathrm{Sr}}_{\mathrm{acceptor}}}\right)}^{\frac{1}{2}}$$

where ${\mathrm{Fe}}_{\mathrm{donor}}$ (${\mathrm{Sr}}_{\mathrm{donor}}$) and ${\mathrm{Fe}}_{\mathrm{acceptor}}$ (${\mathrm{Sr}}_{\mathrm{acceptor}}$) are the concentrations of free Fe (Sr) in the donor and acceptor solutions, respectively. Because the amount of Sr complexing with ligands is negligible, the total concentration of Sr can be taken as the free cation concentration on both sides.

The constant ^c^K (L·g^−1^) is defined as the concentration of metal ions bound to DOM, divided by the concentration of ‘free’ iron in solution, to compare the binding abilities of iron with DOM. The formula is as follows [44]:

(8) K c=[MHS][M]·(HS)

where [MHS] is the concentration of metal ions bound to DOM, mol L^−1^; [M] is the concentration of free metal ions in solution, mol L^−1^; and (HS) is the concentration of DOM in solution, g·L^−1^.

## 3. Results and Discussion

### 3.1. Spectral Properties of Bulk PPFA and Different MW Fractions

#### 3.1.1. UV–Vis Spectroscopy Properties

The characteristics of iron-binding are highly related to the structure and composition of DOM. As shown in Figure S1, the absorbance spectra for both bulk and fractionated PPFA show a decreasing trend as an approximate quasi-exponential change, along with the wavelength in the range of 230–800 nm with a prominent peak at 303 nm. There was an obvious phenomenon that the absorbance of HMW fractions was slightly higher than the bulk PPFA and LMW fractions, suggesting that the molecular weight might affect the phenolic, aromatic carboxylic, and polycyclic aromatic groups. The UV–Vis spectroscopic parameters were employed to further distinguish between the functional groups of these samples, as shown in Table 1. The SUVA_254_ and SUVA_260_ values exhibited a similar trend that increased with the increasing molecular weight of fractionated PPFA. Furthermore, these values are commonly used to refer to aromatic and hydrophobic compounds in DOM, respectively. In contrast, the values of E_250_/E_365_ were negatively correlated with the molecular weight of DOM, with the highest value observed in P4 and the lowest in P1. With respect to the values range of E_250_/E_203_, there were no significant changes associated with the change in molecular weight.

It should be noted that dissolved organic matter (DOM) with larger molecular weight contains more conjugated and unsaturated structures and exhibits more complex structures, which explains why the absorbance of DOM at the same concentration is greater for DOM with larger molecular weight [45]. Moreover, the intramolecular charge transfer ability plays a leading role in the optical properties of DOM, and the decrease in molecular weight will reduce the intramolecular charge transfer potential, making the spectrum shift toward shorter wavelengths [46]. According to the parameters results, HMW fractions possess stronger aromaticity, which is similar to the previously reported values for DOM derived from soil humic acid, because the humification process forms organic components with greater molecular weight, promoting DOM aromatic structure [47]. Meanwhile, HMW fractions contain more hydrophobic fractions than LMW fractions, owing to the more aromatic and aliphatic structures found in higher molecular weight fractions, whereas LMW fractions are rich in hydrophilic carboxyl groups [48,49]. In addition, higher E_250_/E_365_ in low MW fractions of PPFA indicated that the efficiency of the ultrafiltration procedure and E_250_/E_203_ implied almost no difference in the number of substituents on PPFA in each fraction.

#### 3.1.2. 3D EEM Spectroscopy and Fluorescence Indices

To examine the heterogeneities in absorbance between the bulk and fractionated PPFA, fluorescence EEM spectra were additionally measured, which identify the components of DOM based on the positions of emission/excitation maxima. The EEM spectra changes in PPFA for bulk and different MWs are shown in Figures S3 and S4. Two distinct peaks can be observed in the EEM fluorescence, namely peak A (EM/EX: (430–445 nm)/260 nm) and peak C (EM/EX: (430–445 nm)/(325–330 nm)). Peak A and C are respectively stimulated by UV and visible excitation named humic-like fluorophore [50,51]. It was found that the fluorescence intensities of peak A were higher than those of peak C in bulk and fractionated PPFA. Despite the similar contours between different fractions, a slight difference in fluorescence intensity was observed, exhibiting a sequence of P1 > P2 > P3 > P4 > P0 on peak A and peak C, which indicated that the masking effect between fluorescence peaks in bulk PPFA is reduced by fractionation. Based on EEM data, fluorescence indices were calculated to denote the spectral characteristics of MW-dependent samples, as shown in Table 1. FI serves as an indicator of the source of DOM, enabling the differentiation between microbial and terrestrial sources. The FI of LMW-PPFA (1.42) was higher than that of HMW-PPFA (1.26–1.33) and bulk PPFA (1.33). BIX, an indicator of the autochthonous DOM contribution, showed that LMW-PPFA fractions were slightly higher than that of the HMW counterparts. HIX is a determinant of the degree of humification present in DOM samples. In contrast, HIX of high molecular weight fractions was higher than for the low molecular weight ones, as P2 > P1 > P3 > P4, demonstrating that the HMW fractions were characterized by higher HIX than the LMW fractions.

In general, FI with lower values (<1.4) in bulk and HMW PPFA reflected their terrestrial sources. This result is consistent with previous studies in which terrestrial sources are primarily located in high MW fractions and microbial sources dominate the low MW fractions with more autochthonous components [52]. Prior studies have denoted that these terrestrial humic substances contain highly aromatic components, as reported by a negative correlation between aromaticity and FI of humic substances [39,53]. The results of FI were consistent with that of SUV_254_ in UV–Vis spectra, which indicated that the HMW components contained more aromatic compounds. In addition, HIX suggested a higher humification degree for the high MW fractions, in agreement with the E_250_/E_365_ results. Overall, SUVA_254_ and FI jointly indicated that HMW fractions tend to demonstrate terrestrial attributes and possess more aromatic components. SUVA_260_ denoted the stronger hydrophobicity of HMW fractions, while E_250_/E_365_ and HIX conformed the accuracy of fractionation, as well as a greater degree of humification of HMW fractions. Obvious heterogeneities shown by the spectral properties of DOM lead to differences in the ability of metal–DOM complexation, and these distinctions may be more pronounced for different molecular weight fractions [54].

### 3.2. Influence of Different Iron Concentrations on Absorbance and Fluorescence Spectra

#### 3.2.1. Binding Properties on PPFA Absorbance Spectra with Iron

**Figure 1:**
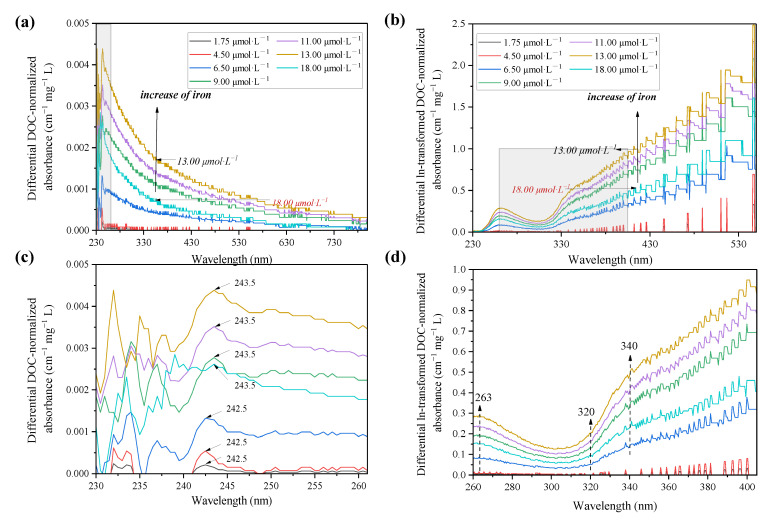
DOC-normalized differential and ln-transformed spectra of the PPFA induced by Fe(III) at varying concentrations. (**b**,**d**) are enlarged details of (**a**,**c**), respectively. The concentration of PPFA is 47.7 mg·L^−1^ and the Fe(III) concentrations range from 1.75 to 18.00 μmol·L^−1^.

The intensity of zero-order absorption spectra decreased quasi-exponentially along with the wavelength range, and changes in the absorbance at all wavelengths in response to Fe(III) addition were consistent but subtly increased, as shown in Figure S2. Nonetheless, these changes were significant and discernible using the differential and ln-transformed differential absorption spectra (Figure 1) [55]. Compared with the zero-order absorption spectra, the differential spectra showed that Fe(III) increased the absorbance of DOM in all wavelengths, and a predominant type of metal-active chromophores with peak maxima at ca. 243 nm is visible in Figure 1b. In response to Fe(III) addition, the differential spectra increased consistently and reached a maximum around 13.00 μmol·L^−1^, while showing a decrease at 18.00 μmol·L^−1^. The phenomenon was also observed in prior studies on the reactions of Fe^3+^ and Al^3+^ with DOM [56]. This indicates that the complexation of metal and DOM leads to an absorbance increase before reaching the critical value, then colloid and co-precipitation occur when the concentration increases.

To reveal the heterogeneity of binding site distribution, the ln-transformed absorbance spectra of PPFA were calculated and exhibited a similar trend compared with the differential spectra, as shown in Figure 1c,d. Several regions with somewhat different slopes are observed in ln-transformed spectra (e.g., <263, 263–309, 309–340, 340–547 and >547 nm), indicating an inconsistent distribution of active sites and functional groups within the PPFA for Fe(III) binding. The addition of Fe(III) induced intensity enhancement for all bands/peaks but with different degrees, in which a distinct characteristic peak was located at 263 nm. The changes in absorbance above ca.320 nm were more significant than the low wavelength band, and the same phenomenon was also found in studies of other heavy metals [35]. In addition, a linear increase in absorbance from 325 nm to 340 nm was observed, with 340 nm as the inflection point, exhibiting a slope from 340 nm to 547 nm, less than that from 325 nm to 340 nm. The prominence observed in the ln-transformed differential spectra at high wavelengths of >320 nm is related to the presence of absorbance bands, which are sensitive to changes in the chemical status of DOM molecules. Therefore, chromophores in the >320 nm region were identified to reflect the iron binding sites. By employing numerical deconvolution of differential spectra of DOM into discrete Gaussian bands, the existence of low-intensity is discovered at ca.380 and 550 nm [35,57]. It can be assumed that these changes were ascribed to the accretion of electric charge and/or other non-specific effects affiliated with DOM deprotonation and metal binding.

#### 3.2.2. Binding Properties on PPFA Fluorescence Spectra with Iron

The 3D EEM fluorescence spectra for PPFA with and without the addition of Fe(III) for various concentrations at pH 2.0 are depicted in Figure S3. Two notable features assigned by Peak A (EX/EM = 260–265 nm/435–455 nm) and Peak C (EX/EM = 330 nm/430–440 nm) were observed in the EEM spectra at all iron concentrations, similar to the results described in prior studies [58]. The peak intensities and locations of fluorophores in the EEM spectra with different Fe(III) concentrations of PPFA are summarized in Table S4. The addition of iron to PPFA only led to intensity quenching, but no obvious change was observed in the shape of the fluorescence landscapes. In order to demonstrate the effects of Fe(III) concentrations on the two fluorescent components, the modified Stern–Volmer model was applied to analyze the fluorescence quenching results. The curves of fluorescence intensity at peak A and peak C versus iron concentration were fitted to obtain the quenching constant, and the corresponding parameters are listed in Table 2. As shown in Figure S5, linear relationships between F_0_/(F_0_ − F) and 1/C_M_ for two peaks when the Fe(III) concentration ranged from 1.75 to 18.00 μmol·L^−1^ proposed a good complexation reaction between PPFA and Fe(III). The logK_M_ value of peak A was 4.90 and 4.72 for peak C, exhibiting a slightly stronger binding affinity for ultraviolet humic-like fluorophore than visible fulvic-like fluorophore for Fe(III).

**Figure 2:**
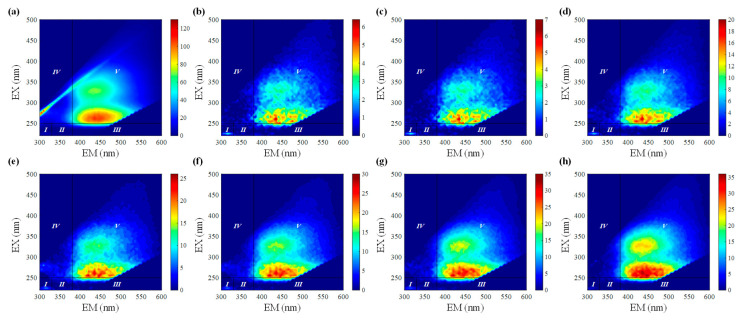
ΔEEMs of PPFA binding with Fe(III) and fluorescence region integration (FRI) results with the addition of iron: (**a**) 0 μmol·L^−1^; (**b**) 1.75 μmol·L^−1^; (**c**) 4.50 μmol·L^−1^; (**d**) 6.50 μmol·L^−1^; (**e**) 9.00 μmol·L^−1^; (**f**) 11.00 μmol·L^−1^; (**g**) 13.00 μmol·L^−1^; (**h**) 18.00 μmol·L^−1^.

**Figure 3:**
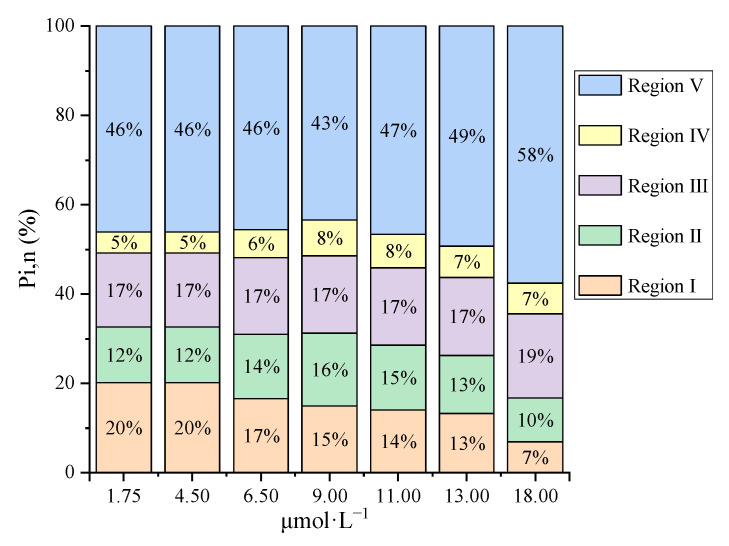
The percentage of contribution of different regions in ΔEEM for bulk PPFA with different iron concentrations.

To quantify the proportion of iron binding with the different fluorophores, the differential fluorescence EEM spectra between the initial bulk PPFA and the PPFA after bound with iron combining FRI analysis were calculated. The ΔEEM landscapes of PPFA after the addition of 1.75, 4.50, 6.50, 9.00,11.00,13.00 and 18.00 μmol·L^−^^1^ of iron, in comparison with those without the addition of iron at pH 2.0, are shown in Figure 2. The fluorescence intensities of PPFA were quenched with increasing iron concentrations in the region of peak A and peak C. More significant quenching was noticed in the peak A fluorescent region and higher concentrations of iron addition. In comparison to the UV-Vis spectra absorbance, the quenching intensity of each peak in EEM fluorescence spectra is still enhanced at 18.00 μmol·L^−1^ concentration. Fluorescence regional integration (FRI) analysis was applied to divide the ΔEEM spectra into five different Ex/Em regions [59]. From Figure 2, the ΔEEM caused by iron mainly focused on Region V (EX/EM 250–450/380–550 nm), indicating that iron significantly quenched the fluorescence intensity in the EEM region associated with humic-like acids. With the addition of iron, P_i,n_ occurring in Region V increased from 46% to 58%, showing higher concentrations generally possessed a stronger binding capacity with humic substances (Figure 3). In comparison with high iron concentrations, it can be found that Region I and Region II occupied relatively larger proportions, indicating that simple aromatic proteins were involved in the binding of Fe(III) and PPFA at low iron concentrations. Previous studies also showed that the contribution of humic acid substances is greater for iron and protein-like substances account for a minor proportion in wetland DOM [40].

### 3.3. Influence of Different PPFA Molecular Weight on Spectral Characteristics

#### 3.3.1. Binding Properties on PPFA Absorbance Spectra with Iron

**Figure 4:**
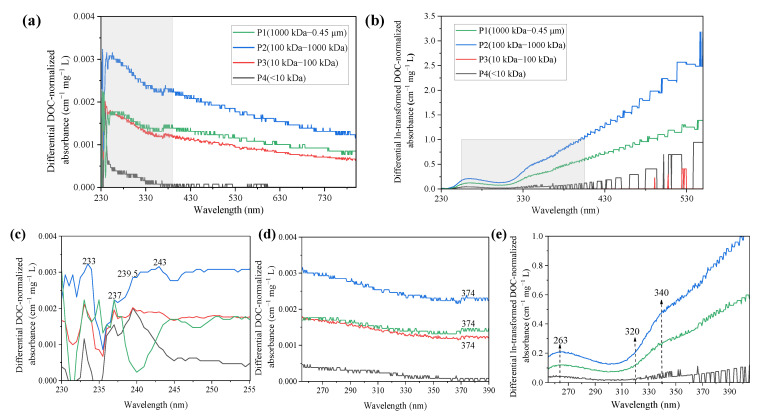
DOC-normalized differential and ln-transformed spectra of the PPFA induced by Fe(III) at varying molecular weight. (**c**,**d**) are enlarged details of (**a**), (**e**) is enlarged details of (**b**). The addition of iron concentrations for P1–P4 were 10.80, 10.07, 10.13 and 10.30 μmol·L^−1^.

The zero-order absorption spectra of different molecular weight fractionated PPFA also displayed featureless characteristics of the binding of PPFA and iron, as shown in Figure S1. The differential and ln-transformed differential absorption spectra at varying PPFA molecular weight are shown in Figure 4. In the wavelength range from 240 to 800 nm, the differential absorbance decreased with the increase in wavelength. Compared with zero-order spectra, iron addition increased the fractionated PPFA absorbance in the following order as P2 > P1> P3 > P4. An obvious characteristic peak at 237 nm occurred in all four MW fractions and a tiny characteristic peak at the wavelength of 374 nm was observed in P1, P2 and P3 but not in P4. Compared with differential spectra obtained from bulk PPFA, a similar significant feature at ca.243 nm was observed but MW-fractionated samples might own a favorable binding site at 374 nm. In addition, the absorbance difference deduced by P4 was smaller than the other three components, revealing that HMW-PPFAs were characterized by stronger binding affinities than the LMW fractions.

The ln-transformed differential absorbance followed the order of P2 > P1> P4 > P3 for PPFA at different molecular weights. In the wavelength range of 230–550 nm, the ln-transformed differential absorption spectra increased gradually with the increase in wavelength, reaching a peak value at 263 nm and then decreasing to a minimum value at 309 nm. For P1 and P2, it could be clearly observed that the absorbance increases after the wavelength of 325 nm, and an obvious inflection point similar to bulk PFA could be observed at the wavelength of 340 nm, showing a significant reaction with the change in molecular weight, where the >320 nm region represents active binding sites, a conclusion consistent with the performance of bulk PPFA in Figure 1d. A broad peak occurred at 335 nm has been identified as a response to Fe(III) addition for DOM originated from the Beidagang Wetland in the previous study [40].

#### 3.3.2. Binding Properties on PPFA Fluorescence Spectra with Iron

As shown in Figure S4, the fluorescence EEM spectra for different molecular weight PPFA fractions also showed two prominent peaks, similar to bulk PPFA. The PPFA fluorescence at different molecular weight showed different degrees of quenching after complexing with different metals. To figure out the consistency and difference of fluorophores in different molecular weight fractions, the peak intensities and peak locations of fluorophores in the EEM spectra of fractionated PPFA are summarized in Table S5. The peak locations of excitation wavelengths for these two fluorophores were near 260 and 330 nm, respectively.

**Figure 5:**
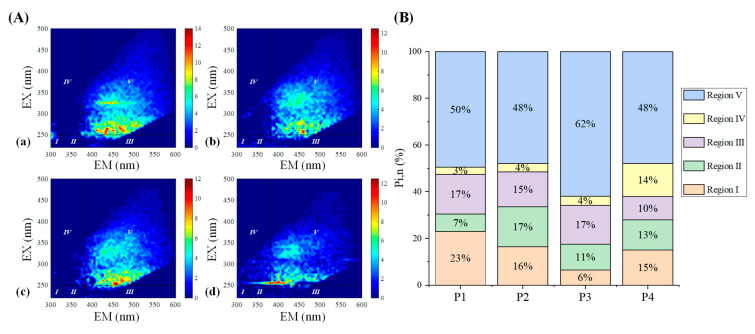
(**A**) ΔEEMs of different MW PPFA fractions binding with Fe(III) and fluorescence region integration (FRI) results with the addition of iron. (**a**) P1; (**b**) P2; (**c**) P3; (**d**) P4. (**B**) The percentage of contribution of different regions in ΔEEM for different MW PPFA fractions.

The ΔEEMs caused by Fe(III) from P1 to P4 have a similar contour, as shown in Figure 5, with two fluorophores appearing in their humic acid-like regions, suggesting that Fe(III) bound with the same kinds of fluorescent components at different molecular weight fractions. From P1 to P3, the strongest quenched fluorescence peaks were all located in region V, with excitation wavelengths of 260–265 nm and an emission wavelength of 435 nm, while the proportion of fluorescence quenched intensity in region I and region II were various. Concerning P4, despite those quenched fluorophores being located in region V, the highest fluorescence peak in this region also appeared at the excitation wavelength of 270 nm and the emission wavelength of 400 nm. As summarized in Figure 5, there is little difference in the proportion of fluorescent groups quenched by Fe(III) for P1 to P3 in region V. In addition, the percent fluorescence response of soluble microbial by-product-like components in region I declined, implying simple aromatic proteins were involved in the binding of Fe(III) and PPFA at high MW fractions. However, about 62% of humic-like substances were involved in the binding of Fe(III) and P3, indicating humic-like substances play a more significant role in P3 and Fe(III) binding. 

### 3.4. Effect of PPFA on Fe Speciation

#### 3.4.1. The Speciation of Iron in Different Concentrations

The chemical speciation of iron after interacting with different concentrations is described in Table 3. The recoveries of iron in each set of experiments were 94.6–98.3%. In the donor solution, the initial Fe concentrations were 253–1021 μg·L^−1^ (4.50–18.00 μmol·L^−1^). The Fe concentrations measured in the acceptor solution were 124–747 μg·L^−1^ (2.21–13.34 μmol·L^−1^), in accordance with the corrected ‘free’ Fe concentration from 105 to 789 μg·L^−1^ (1.88–14.09 μmol·L^−1^). The iron concentrations expressed in μg·L^−1^ are discussed in the following text. The proportion of bound Fe complexes varied from 22.7% to 58.5%, which decreased with the increase in the total Fe concentration (R^2^ = 0.8761). As shown in Table 3, the range of ^c^K was 6.2–29.5 L·g^−1^, which also decreased with the increase in total Fe concentration. As a consequence, the ^c^K value of the P0 (I) group was significantly higher than that of the other three groups, indicating that high-affinity sites in DOM were preferentially coupled to Fe at low concentrations. The high-affinity sites tend to be saturated as the Fe concentration increases, while the low-affinity sites begin to react with Fe, resulting in a weaker interaction between the metal ions and DOM [60]. The little difference in ^c^K values between P0 (III) and P0 (IV) groups may be explained by the saturation of binding sites, as illustrated by the UV–Vis spectra analysis. It was also found that the binding capacity increased with the increase in iron concentration, which is highly consistent with the changes in fluorescence spectroscopy. The more Fe complexing with DOM, the higher the degree of fluorescence quenching, which is consistent with the results of previous studies [61].

#### 3.4.2. The Speciation of Iron in Different PPFA Molecular Weight

Table 4 displays the difference in iron speciation on different molecular weight fractionated PPFA. In the donor solution, the initial Fe concentrations were 605, 564, 567 and 577 μg·L^−1^, corresponding to 10.80, 10.07, 10.13 and 10.30 μmol·L^−1^. In the four groups of experiments, the recovery of Fe ranged from 94.2 to 96.8%. The proportion of bound Fe was between 25.3 and 39.8%, and the range of ^c^K value was 6.6–13.7 L·g^−1^, which decreased with the decrease om molecular weight. The diverse properties caused by the molecular weight of DOM greatly affected the way and degree of binding with heavy metals. The higher molecular weight DOM normally has the stronger metal complexing ability, because of higher humification and aromaticity, stronger electrostatic field and it is easier to form poly-dentate metal complexes [62]. In addition, there are more oxygen-containing functional groups that strengthen the complexation ability of HMW DOM binding to metals [52]. Previous studies on the binding of copper and mercury to DOM in water samples, including rivers and swamps, have also shown a stronger binding capacity for HMW [59]. Nevertheless, it is not completely clear how the molecular weight affects the complexation between the metals and DOM complexes, due to the variation in environment and metal.

#### 3.4.3. Correlation between PPFA Composition and ^c^K

**Figure 6:**
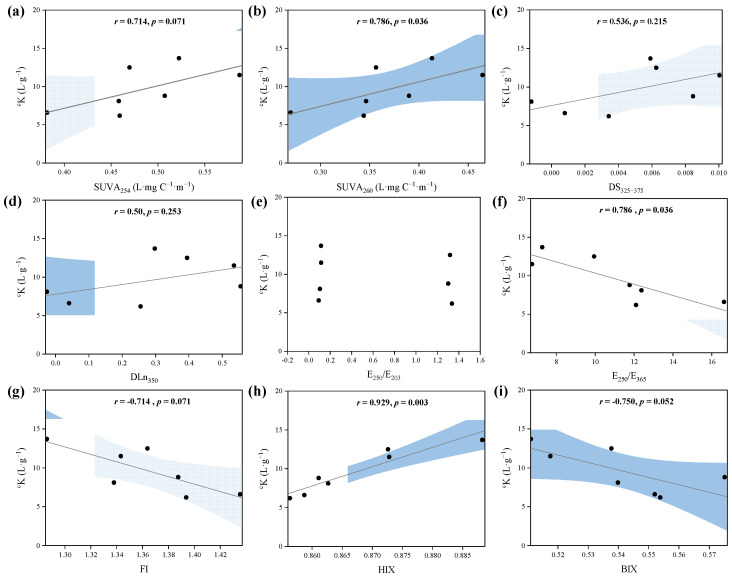
Spearman’s rank correlations between cK and PPFA optical parameters, including (**a**) specific UV absorbance at 254 nm (SUVA_254_), (**b**) specific UV absorbance at 260 nm (SUVA_260_), (**c**) DS_325–375_, (**d**) DLn_350_, (**e**) E_250_/E_203_ (**f**) E_250_/E_365_, (**g**) fluorescence index (FI), (**h**) humification index (HIX) and (**i**) biological index (BIX).

After the removal of iron at 253 μg·L^−1^ from the entire dataset, the correlations showed the obvious correlation between ^c^K and bulk and fractionated PPFA spectral property indices, as presented in Figure 6. ^c^K indicated significant correlations with SUVA_254_ and SUVA_260_, indicating the binding strength of PPFA–Fe(III) increased with increasing aromaticity and hydrophobicity. Similar relationships have also been observed in the binding between DOM and other metals [63,64]. Aromatic humic substances are considered the controlling metal chelators, and the strong positive correlation between Fe(III) and aromatic carbon content was verified in natural freshwater [63,65,66]. Due to oxygen-containing functional groups, the hydrophobic acid fraction of DOM can promote the stability of complexes through π-interactions, exhibiting a strong binding affinity for metals, such as Fe and Al [25,67]. Additionally, DS_325_–_375_ and DLn_350_ presented a relatively weak positive correlation trend with ^c^K, which may support that the affinity of Fe(III) binding to PPFA was associated with the differential spectra and specific spectral slopes based on UV–Vis spectra. Furthermore, the negative relationship between ^c^K and E_250_/E_365_ revealed that the stronger binding affinity of higher molecular weight PPFA E_250_/E_203_ was not correlated with ^c^K, indicating that this parameter is not sensitive to reflecting the binding with Fe(III). Terrestrial-dominant properties of PPFA (with lower FI value) showed greater binding ability, supported by the negative correlation between ^c^K and FI. Previous studies have shown that terrestrial sources of PPFA potentially provide more binding sites because of more hydrophobic fractions and higher molecular weight [54]. The negative relationship between BIX and ^c^K explained that autochthonous DOM has low binding strength. In all the selected parameters, HIX showed the strongest correlation between Fe(III) and ^c^K, indicating that the humification degree of PPFA played a great role in binding strength. The degree of humification was an important indicator of peat soil, which could be used to represent the degree of peat decomposition and the conversion of fresh organic matter to recalcitrant humic substances.

Additionally, it should be noted that not all spectral parameters could indicate the reactivities of DOM binding with Fe(III). Overall, the UV–Vis parameter, such as SUVA_254_, SUVA_260_, E_250_/E_365,_ combined with FI, BIX and HIX, could be used as effective indicators to predict the binding strength of PPFA with Fe(III), especially the HIX corresponding to the degree of humification, which can well characterize the binding strength between peatland DOM and iron.

## 4. Conclusions

In this study, the binding characteristics of Fe(III) to Pahokee Peat fulvic acid (PPFA) were investigated using differential and ln-transformed differential absorption spectra, 3D EEM combined with FRI analysis, and DMT analytical techniques. The chemical structure and functional group composition of HMW PPFA differed from bulk and LMW PPFA. HMW PPFA contained higher SUVA_254_, SUVA_260_ and HIX, but lower E_250_/E_365_, FI and BIX than bulk and LMW PPFA. Differential spectra and ln-transformed differential absorption spectra revealed that specific binding sites for iron reflected in the absorption spectra were mainly located at the 263 nm and >320 nm regions. The 3D EEM analysis revealed the different degrees of binding of iron and fluorescence components in bulk and fractionated PPFA. In the process of iron addition in PPFA, humic-like components were discovered to be the principal quenching components and ultraviolet humic-like fluorophores had a higher binding affinity than visible fulvic-like fluorophores. The results of the spectral analysis exhibited greater binding capacity with the increase in iron concentration. As for the MW-fractionated samples, the HMW-PPFA (>10 kDa) exhibited higher metal binding potential than the bulk and LMW counterparts. The change in iron speciation was analyzed by DMT, in which the concentration of bound iron corresponded to the spectroscopy results. Moreover, the complexation characteristics were further explained by the correlation between the equilibrium constant ^c^K and the spectral parameters. The correlation demonstrates that the presence of oxygen-containing functional groups, high aromaticity, hydrophobicity and terrestrial attribute will make PPFA more capable of binding to iron. Moreover, the degree of humification is highly correlated with ^c^K, and HIX can be used as a significant indicator for the complexation of peatland-derived DOM and Fe. This work further investigates the mechanism of the reaction of iron and peat-DOM and may show a way to quantify this iron in different regions.

## Figures and Tables

**Table 1:** UV–Vis absorption and fluorescence spectral parameters of bulk PPFA (P0) and fractionated PPFA (P1–P4).

Parameters	P0	P1	P2	P3	P4
SUVA_254_	0.405	0.510	0.475	0.492	0.347
SUVA_260_	0.296	0.394	0.360	0.372	0.244
E_250_/E_365_	13.458	9.947	11.774	12.100	17.167
E_250_/E_203_	0.094	0.109	0.105	0.105	0.090
FI	1.33	1.26	1.33	1.31	1.42
BIX	0.508	0.50	0.52	0.52	0.55
HIX	0.8924	0.8871	0.8883	0.8831	0.8687

**Table 2:** The initial proportion and the conditional stability constant of each fluorophore in PPFA.

Peak	Fraction	logK_M_	f	R^2^
Peak A	Ultraviolet humic-like	4.90	47.8	0.9796
Peak C	Visible humic -like	4.72	53.5	0.9918

**Table 3:** Results of chemical speciation of iron in the solutions of PPFA at different iron concentrations.

	[Fe^3+^]_initial_ (μg·L^−1^)	Measured [Fe^3+^ ]_96h_^a^ (μg·L^−1^)		Corrected [Fe^3+^]_acceptor_ (μg·L^−1^)	Iron Bound to PPFA Fraction (%)	^c^K (L·g^−1^)	Binding Capacity (g·kg^−1^)	Recovery (%)
	Donor	Donor	Acceptor					
P0(I)	253	232	124	105	58.5	29.5	3.10	96.2
P0(I)	517	482	234	324	37.3	12.5	4.05	96.8
P0(III)	749	689	546	527	29.6	8.8	4.65	95.9
P0(IV)	1021	959	747	789	22.7	6.2	4.86	99.2

^a^ The Donnan equilibrium was reached after 96 h.

**Table 4:** Results of chemical speciation of iron in the solutions of PPFA with different molecular weight.

	[Fe3^+^]_initial_ (μg·L^−1^)	Measured [Fe3^+^]_96h_^a^ (μg·L^−1^)		Corrected [Fe^3+^]_acceptor_ (μg·L^−1^)	Iron Bound to PPFA Fraction (%)	^c^K (L·g^−1^)	Binding Capacity (g·kg^−1^)	Recovery (%)
	Donor	Donor	Acceptor					
P1	605	563	325	364	39.8	13.74	5.05	96.2
P2	564	512	321	361	36.0	11.45	4.26	94.2
P3	567	523	429	412	27.3	8.09	3.25	96.8
P4	577	535	372	431	25.3	6.60	3.06	96.6

^a^ The Donnan equilibrium was reached after 96 h.

## Data Availability

The data presented in this study are available upon request from authors.

## Supplementary Materials

The following supporting information can be downloaded at: https://www.mdpi.com/article/10.3390/ijerph19137838/s1, Figure S1: UV-Vis absorbance spectra of bulk and fractionated PPFA; Figure S2: UV-Vis absorbance spectra of PFA with different Fe concentrations; Figure S3: 3D EEM of bulk PPFA with different Fe concentrations; Figure S4: 3D EEM of PPFA with different molecular weight before and after interaction with Fe(III); Figure S5: Modified Stern-Volmer plots for the fluorescence quenching of PPFA by Fe(III) on peak A and peak C; Table S1: IHSS-quantified chemical characterization data of the Pahokee Peat fulvic Acid II (PPFA, 2S103F); Table S2: The percentage content of all grades of PPFA; Table S3: The concentration of experimental substances in the interaction between bulk and fractionated PPFA and Fe; Table S4: Locations and intensities of peaks in EEM spectra of PPFA with different Fe concentrations; Table S5: Locations and intensities of peaks in EEM spectra of PPFA with different molecular weights; Table S6: Measured and corrected Fe concentrations at the acceptor side for different Sr concentrations at the acceptor side and a constant Sr concentration at the donor side using DMT results after 96 h.
