# Supplementary material for: Spectroscopic Characteristics and Speciation Distribution of Fe(III) Binding to Molecular Weight-Dependent Standard Pahokee Peat Fulvic Acid
Source: Int J Environ Res Public Health. 2022 Jun 26;19(13):7838. doi: 10.3390/ijerph19137838 (PMC9266197; doi:10.3390/ijerph19137838)
Supplement: Supplementary file 1 [file ijerph-19-07838-s001.zip › ijerph-1764363-supplementary.pdf]

Table S1. IHSS-quantified chemical characterization data of the Pahokee Peat fulvic Acid II (PPFA, 2S103F).

Element/compound	%(w/w)
H_2O	9.3[a]
Ash	0.90[b]
C	51.31[c]
H	3.53 [c]
O	43.32 [c]
N	2.34 [c]
S	0.76 [c]
P	<0.01 [c]

[a] H_2O content is the %(w/w) of H_2O in the air-equilibrated sample (a function of relative humidity).

[b] Ash is the %(w/w) of inorganic residue in a dry sample.

[c] C, H, O, N, S, and P are the elemental composition in %(w/w) of a dry, ash-free sample.

Table S2. The percentage content of all grades of PPFA.

DOM	Molecular weight (MW)	Proportion (%)
P1	1000 kDa-0.45μm	56.4
P2	1000 kDa	9.2
P3	10-100 kDa	12.5
P4	<10 kDa	21.9

Table S3. The concentration of experimental substances in the interaction between bulk and fractionated PPFA and Fe.

DOM	DOC ($mg \cdot L^{-1}$)	Fe ($\mu mol \cdot L^{-1}$)
		1.75
		4.50
		6.50
Bulk PPFA(P0)	47.7	9.00
		11.00
		13.00
		18.00
P1	48.2	10.80
P2	49.1	10.07
P3	46.5	10.13
P4	51.3	10.30

Table S4. Locations and intensities of peaks in EEM spectra of PPFA with different Fe concentrations.

Iron concentration (μmol·L^{-1})	Peak A		Peak C	
	EX/EM(nm)	Intensity	EX/EM(nm)	Intensity
0	260/435	108.92	330/435	71.39
1.75	260/445	102.31	330/430	68.38
4.50	260/445	96.70	330/430	64.12
6.50	260/445	93.07	330/430	61.51
9.00	260/445	87.01	330/440	57.99
11.00	260/445	82.44	330/440	54.55
13.00	265/435	76.02	330/430	51.39
18.00	265/455	73.69	330/440	47.35

Table S5. Locations and intensities of peaks in EEM spectra of PPFA with different molecular weights.

Fraction	Peak	DOM		DOM+Fe	
		EX/EM(nm)	Intensity	EX/EM(nm)	Intensity
P0	A	260/435	108.92	260/445	87.01
	C	330/435	71.39	330/440	57.99
P1	A	260/435	133.30	260/440	126.10
	C	325/445	96.16	330/440	86.80
P2	A	260/445	130.54	260/435	125.40
	C	330/435	91.19	330/435	86.20
P3	A	260/445	120.97	265/445	114.40
	C	325/430	84.66	330/435	81.26
P4	A	260/445	113.60	260/435	107.50
	C	330/430	81.93	330/430	76.32

Table S6. Measured and corrected Fe concentrations at the acceptor side for different Sr concentrations at the acceptor side and a constant Sr concentration at the donor side using DMT results after 96 h.

$[Sr]_{initial}$ (μmol·L^{-1})		Measured $[Sr]_{96h}$ (μmol·L^{-1})		Measured $[Fe]_{96h}$ (μmol·L^{-1})		Corrected [Fe] (μmol·L^{-1})
Donor	Acceptor	Donor	Acceptor	Donor	Acceptor	Acceptor
10	2	9.63	2.36	11.69	1.4	11.51
10	4	9.74	4.39	10.51	3.21	10.63
10	6	9.87	6.21	10.37	5.12	10.26
10	10	10.02	9.85	10.25	9.91	10.17

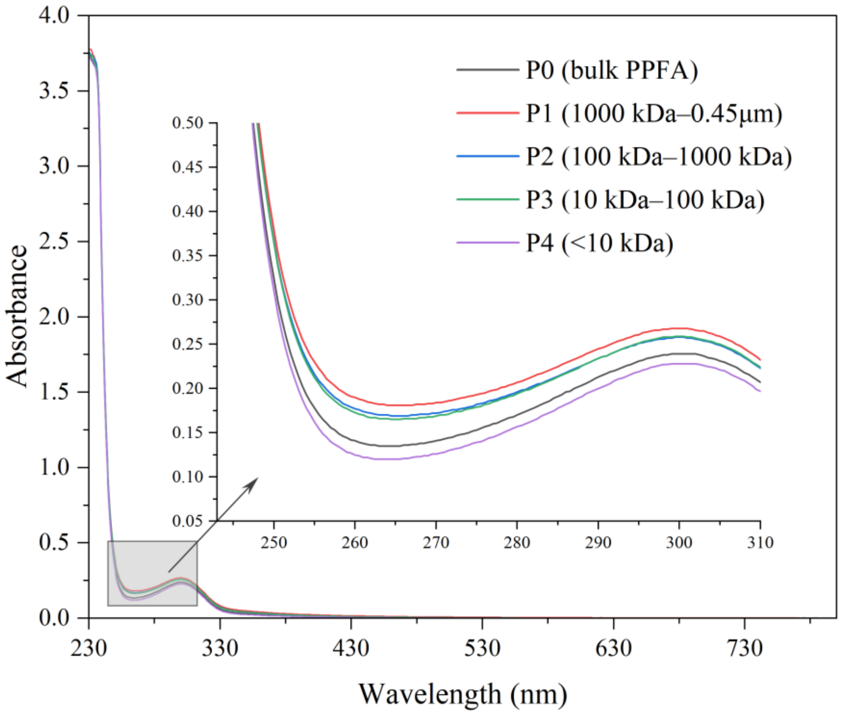

Figure S1. UV-Vis absorbance spectra of bulk and fractionated PPFA.

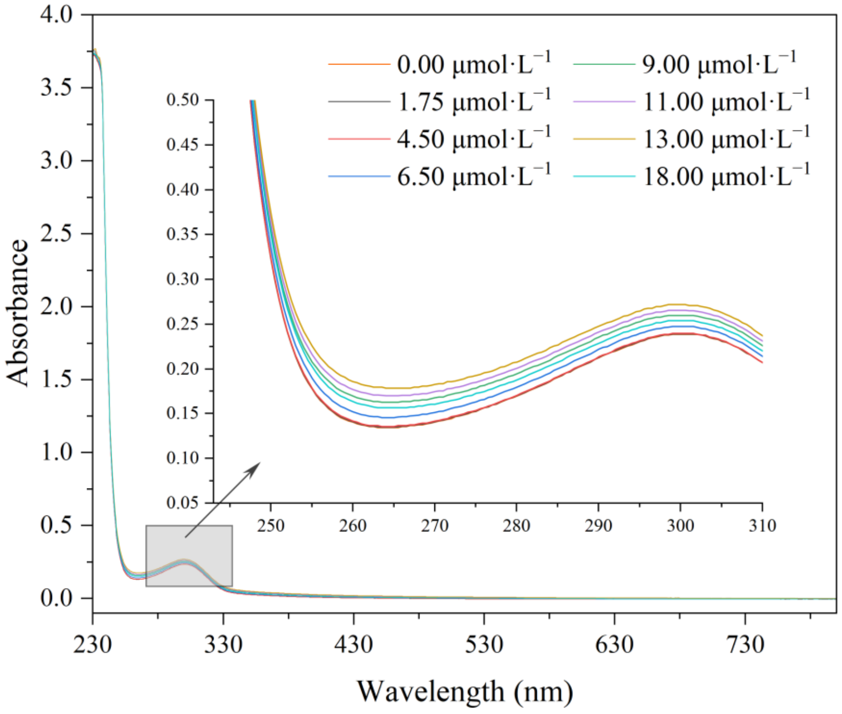

Figure S2. UV-Vis absorbance spectra of PFA with different Fe concentrations.

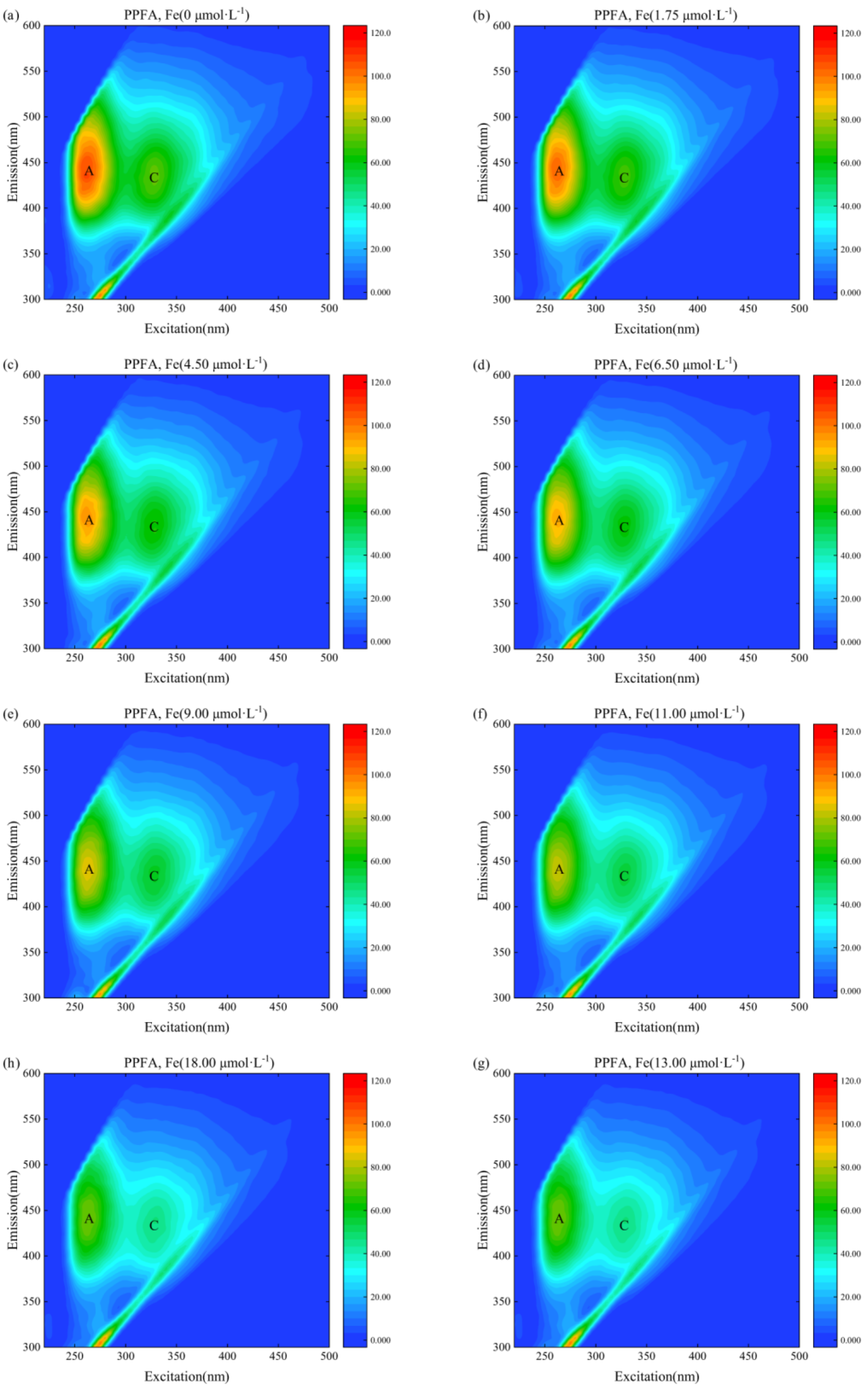

Figure S3. 3D EEM of bulk PPFA with different Fe concentrations.

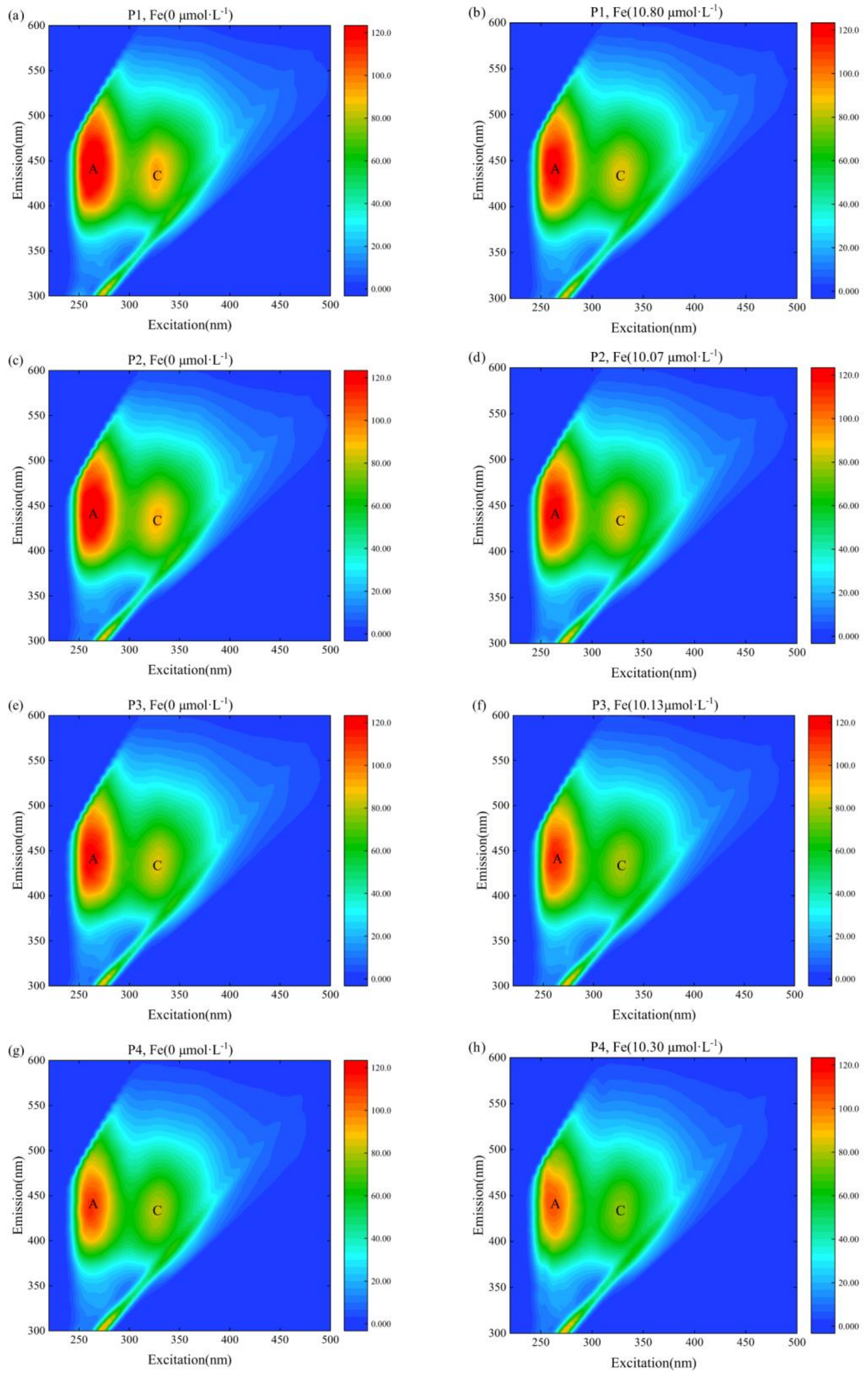

Figure S4. 3D EEM of PPFA with different molecular weight before and after interaction with Fe(III).

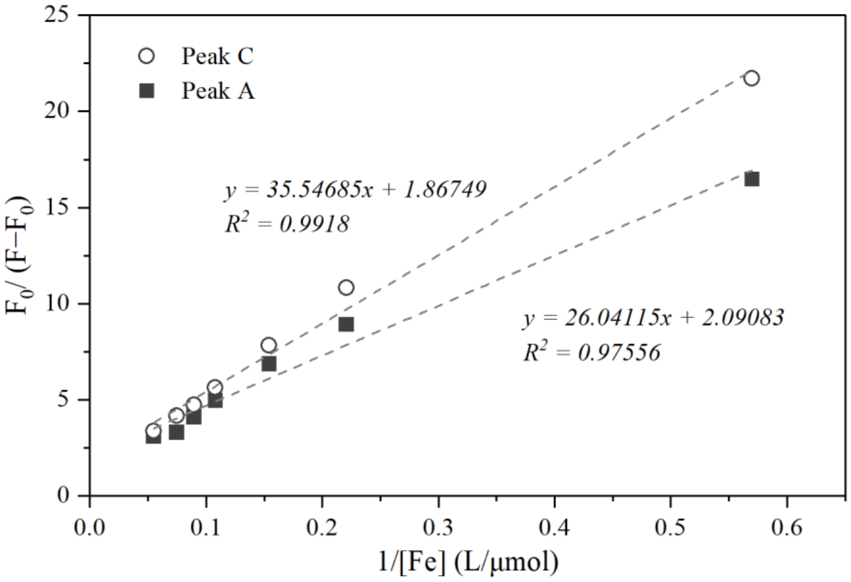

Figure S5. Modified Stern-Volmer plots for the fluorescence quenching of PPFA by Fe(III) on peak A and peak C.
